# The Use of Point-of-Care Ultrasound (POCUS) in Anesthesiology: A Narrative Review

**DOI:** 10.7759/cureus.70039

**Published:** 2024-09-23

**Authors:** Rutuja Gohad, Sudha Jain

**Affiliations:** 1 Department of Anesthesiology, Jawaharlal Nehru Medical College, Datta Meghe Institute of Higher Education and Research, Wardha, IND

**Keywords:** abdominopelvic ultrasound, airway management, anesthesiology, cardiac ultrasound, lung ultrasound, patient care, perioperative management, pocus

## Abstract

Point-of-care ultrasound (POCUS) has become an essential tool in modern anesthesiology, offering real-time imaging that aids in diagnosis, treatment, and procedural guidance. Its applications have expanded significantly, from routine assessments to complex perioperative management, enhancing patient care across various domains. This study aimed to review and summarize the current literature on the applications of POCUS in anesthesiology, focusing on its utility in airway management, cardiac assessment, lung ultrasound (LUS), and abdominopelvic evaluations. Additionally, it discusses the challenges and strategies for overcoming barriers to its widespread adoption. A narrative literature review was conducted, encompassing studies on using POCUS in anesthesiology. Key databases were searched for relevant articles ("Point-of-care ultrasound," "Focused cardiac ultrasound," "Lung ultrasound*"*)*, *and studies were selected based on their relevance to the main topics of interest: airway management, cardiac ultrasound, LUS, and abdominopelvic and gastric ultrasound. Both observational studies and randomized controlled trials were included. POCUS improves the safety and accuracy of regional anesthesia and airway procedures, enhancing patient outcomes by providing real-time visualization. Focused cardiac ultrasound is valuable in preoperative, intraoperative, and postoperative care, allowing for tailored anesthesia plans and timely interventions based on cardiac function. The US is an effective diagnostic tool for assessing pulmonary conditions, with specific signs and artifacts aiding in identifying pathologies such as pneumothorax, pleural effusions, and pulmonary congestion. Despite its benefits, POCUS faces barriers such as limited access to training, the need for standardized protocols, and the high equipment cost, especially in low-resource settings. While its integration into clinical practice continues to grow, overcoming challenges related to training, standardization, and equipment access is essential to realize its full potential. Continued efforts in education and research are needed to support the widespread adoption of POCUS in anesthesiology.

## Introduction and background

For more than 50 years, doctors have used ultrasonography, a comparatively safe, portable, affordable, and easily accessible imaging method, for diagnostics [1]. Point-of-care ultrasound (POCUS) is the concept of performing imaging studies immediately at the patient's bedside, which helps in the assessment, management, and intervention that may be required. POCUS should be used in general when it can be used to accomplish particular objectives in a short time and when the patients' outcome improves correspondingly. The POCUS is just one of the many assessment techniques employed in routine patient examinations, and it has also experienced a remarkable transformation within the past few years. These ultrasound devices have become smaller, cheaper, and capable of achieving higher image quality, which has increased the use of such equipment during physicians' perioperative patient evaluations [2]. Bedside ultrasound is currently the standard of care in anesthesiology for nerve blocks and vascular access. However, there has been an increasing interest in how to incorporate whole-body POCUS into the daily practice of contemporary anesthesiologists. This includes but is not limited to using it for procedural purposes and supplementing traditional assessment and management of the patient's care [3].

High-frequency sound waves above human hearing, or more than 20,000 Hz (20 kHz), are referred to as ultrasounds. Diagnostic ultrasound operates at millions of hertz, with lower frequency probes typically operating between 2 and 5 MHz and higher frequency probes operating between 6 and 13 MHz [1]. The three most popular varieties are phased array, linear, and curvilinear probes. The frequency of each probe varies, affecting image resolution but having an inverse relationship with probe penetration depth. Although the high frequency of linear probes enables superior resolution of surface structures, the low penetration prevents the visualization of deeper structures. Thus, linear probes work best for superficial structures like vascular access or a more thorough assessment of the lung pleura.

On the other hand, curvilinear and phased array probes operate at lower frequencies. Still, they penetrate deeply, making it possible to see deeper structures, like those for the abdomen and heart examinations. The probe on the phased array has a smaller footprint than the curved probe, making it simpler to get sufficient views between the ribs when performing cardiac or pulmonary examinations [4]. Additionally, a growing number of recent clinical research studies have shown that, in comparison to other initial bedside assessment tools, ultrasound is more cost-effective and has better diagnostic sensitivity and specificity [2]. POCUS is a more effective initial diagnostic tool for the cardiovascular assessment of non-ST segment elevation chest pain than the electrocardiogram (EKG). POCUS offers immediate visualization of the heart's anatomy and function, allowing clinicians to identify wall motion abnormalities, estimate ejection fraction, and assess for pericardial effusion. This capability not only aids in the diagnosis of acute coronary syndromes but also facilitates the detection of other potential causes of chest pain, such as pulmonary embolism or aortic dissection [5]. Furthermore, POCUS is more accurate in detecting main stem bronchial intubation than chest auscultation [6]. POCUS is more effective, faster, and more accurate for detecting pneumothorax, pleural effusions, and consolidation in critical care settings than chest auscultation or chest radiograph [7].

## Review

Applications of POCUS in anesthesia

Airway Management

A review of the literature was conducted by Hurtado et al. to assess the function of POCUS in regional anesthesia for airway management. According to the author, POCUS is used to evaluate the upper airway, which is important for efficient airway care. This entails recognizing anatomical landmarks and making sure that instruments are properly visible. It also highlights the importance of visualizing the needle placement during airway blocks in real time. This method improves patient outcomes by increasing the accuracy of regional anesthetic techniques. Specific methods for performing translaryngeal blocks are described, such as using a linear probe with a focus at 1 cm and a depth setting of 3-4 cm. Conclusions drawn from the paper emphasize the significance of POCUS in improving the safety and efficacy of regional anesthesia and airway management, advocating for individualized, less-invasive techniques while highlighting the necessity of proper training and anatomical knowledge [8].

Arumugam et al. reviewed the literature to discuss the application of POCUS in regional anesthesia and its broader implications in perioperative care. The paper emphasizes the analysis of POCUS applications specifically related to airway management, assessment of gastric contents, and trauma evaluation. The authors found that integrating POCUS into regional anesthesia practice is a significant advancement that can improve patient care. POCUS can help avoid delays or cancellations of surgeries by facilitating immediate and accurate assessments of airway anatomy. With real-time imaging, anesthesiologists can identify potential challenges related to airway structure, such as anatomical variations or obstructions, before the induction of anesthesia. This proactive assessment allows for early planning of alternative strategies, such as specialized intubation techniques, which can be crucial in managing difficult airways. This capability can lead to improved operational efficiency and better patient experiences. The paper concludes with a call for more anesthesiologists to embrace POCUS in their practice [9].

Wu and Li did an observational study that advocates for using POCUS as a fast, convenient, and noninvasive alternative to traditional imaging methods such as computed tomography (CT), magnetic resonance imaging, and conventional ultrasound. It enables immediate bedside assessments, reducing wait times and minimizing patient discomfort compared to these more invasive or time-consuming approaches. POCUS can effectively visualize the placement of bronchial blockers (BBs) by detecting ultrasonic signs indicative of one-lung ventilation. The study concludes that POCUS is a promising and effective method for confirming the placement of BBs in pediatric patients. It highlights that POCUS can provide real-time visualization of lung isolation, which is crucial for ensuring the safety and efficacy of one-lung ventilation during thoracoscopic surgeries [10].

POCUS in anesthesia enhances clinical decision-making, particularly in endotracheal tube (ETT) placement. Anesthesiologists utilize POCUS to assess ETT positioning airway size and to predict the appropriate diameter of ETTs, ensuring accurate placement and reducing the risk of complications [11,12]. POCUS can differentiate between tracheal, esophageal, and endobronchial intubation, aiding in preventing airway-related issues. Additionally, ultrasound of the upper airway can accurately locate the cricothyroid membrane for emergency airway access and identify tracheal rings for ultrasound-guided tracheostomy, further improving patient safety and procedural success. Using POCUS in ETT placement showcases the versatility and effectiveness of ultrasound technology in optimizing anesthesia practices and patient outcomes [13].

Cardiac (Focused Cardiac Ultrasound)

Pallesen et al. did a randomized controlled trial to investigate the effects of preoperative focused cardiac ultrasound (FOCUS) in high-risk patients undergoing noncardiac surgery. The study focused on high-risk patients scheduled for noncardiac surgery. Participants in the intervention group underwent preoperative FOCUS. This technique provides valuable information about the cardiac status of patients and can reveal previously unknown cardiac issues. The ultrasound findings were used to tailor the anesthesia plan for each patient, potentially improving outcomes. The primary outcomes measured were the proportion of patients admitted to the hospital for more than 10 days or who died within 30 days after surgery. The study hypothesized that preoperative FOCUS would lead to a decrease in the number of patients who were either admitted to the hospital for more than 10 days or who died within 30 days after surgery. By utilizing FOCUS, the treatment and anesthesia plans could be customized based on the cardiac status of each patient [14].

Numerous studies have demonstrated the effectiveness and practicality of integrating FOCUS into preoperative, intraoperative, and postoperative care for elective and emergency cases. This technique offers critical insights that can substantially impact patient management [15]. It enables rapid assessment of biventricular function, detection of gross valvular abnormalities, identification of pericardial effusions, recognition of wall motion abnormalities indicative of ischemia, and evaluation of a patient's volume status through measures like inferior vena cava collapsibility and ventricular size. There are multiple reports of bedside cardiac ultrasound proving invaluable for prompt diagnoses and adjustments in intraoperative management. For example, it has been used to detect left ventricular inferior wall akinesis in a patient who experienced sudden, severe hypotension after a procedure, even in the absence of EKG changes that would typically indicate acute ischemia [16].

Sheth et al. reviewed the study that evaluated the impact of FOCUS on management decisions. It was noted that in 94% of cases, there was a change in management based on the findings from FOCUS. This assessment was crucial in determining how FOCUS influenced clinical outcomes. The study included the use of FOCUS both intraoperatively and postoperatively. This allowed for the real-time evaluation of patients' conditions and facilitated timely interventions based on the ultrasound findings. The study concludes that FOCUS is an excellent clinical adjunct during the perioperative period. It enhances the anesthesiologist's ability to make informed management decisions, particularly for patients undergoing noncardiac surgery who may be at higher risk due to underlying cardiovascular conditions. A critical finding is that FOCUS led to a change in management in 94% of the cases examined. This indicates that using FOCUS significantly influences clinical pathways and can improve patient outcomes by allowing for timely interventions based on ultrasound findings [17]. A phased array probe is well suited for this type of examination due to its lower frequency and deeper penetration, which allow for effective visualization of cardiac structures while maintaining a small "footprint" for accessing intercostal windows. The heart's oblique position within the chest, surrounded by ribs and lungs that ultrasound waves cannot penetrate, can make obtaining clear images challenging. While patients can be examined in the supine position, the left lateral decubitus position is preferable for parasternal and apical views, as it brings the heart's structures closer to the chest wall for better imaging [18].

Lung Ultrasound

Basumatary et al. did an observational study on 70 patients. Only those undergoing elective extrathoracic surgeries lasting over three hours under general anesthesia were selected for the study. A preoperative, intraoperative, and postoperative lung ultrasound (LUS) was performed on all patients to establish a baseline for lung conditions before surgery. The study monitored the volume of intraoperative fluid administered to each patient and the appearance of three or more "B" lines on the ultrasound, which was used as the criterion for diagnosing lung congestion. The findings emphasize the need for careful fluid management during surgeries, particularly those of longer duration, to minimize the risk of postoperative pulmonary congestion. The study advocates for using LUS as a noninvasive method for early detection of pulmonary congestion, which can help guide postoperative care [19].

Bedside LUS for evaluating pulmonary diseases is not a new technique, having been employed in critical care since the early 1990s [20]. It later gained traction in emergency medicine and, more recently, in perioperative settings by anesthesiologists. LUS is a valuable, efficient, and precise goal-directed diagnostic tool for real-time assessment of respiratory symptoms. Due to the high ultrasound reflectivity at the air-tissue interface, air-filled lungs cannot be directly visualized, making it impossible to assess normal pulmonary parenchyma with ultrasound. Interestingly, this limitation is diagnostically advantageous, as LUS depends on recognizing specific signs, artifacts, and patterns linked to certain pathologies, which are distinct and easily identifiable. A high-frequency linear probe, which can visualize structures up to 4 cm deep, is ideal for examining the superficial pleural line and lung parenchyma. It provides the best resolution for assessing abnormal pleural surfaces, making it the preferred probe for evaluating conditions like pneumothorax or edema. Lower frequency probes are better suited for examining deeper, subpleural artifacts, such as effusions [21].

Abdominopelvic and Gastric Ultrasound

Focused assessment with sonography for trauma (FAST) plays a crucial role in abdominopelvic anesthesia by aiding in the rapid and accurate detection of intra-abdominal injuries, especially in trauma patients. Studies have shown that FAST has reasonable sensitivity, specificity, and accuracy in detecting free fluid in traumatic renal injuries [22]. Additionally, FAST has been widely accepted and integrated into trauma management protocols, demonstrating high specificity and negative likelihood ratio in pediatric patients with blunt torso trauma [23]. Its use allows for the timely identification of intra-abdominal pathology, potentially reducing the need for more time-consuming imaging modalities like CT. Understanding the diagnostic accuracy of FAST, including its strengths and limitations, is essential for informed decision-making and optimal patient management in abdominopelvic anesthesia [24].

The use of POCUS in the FAST procedure has been effectively incorporated into emergency medicine, critical care, and surgery. Rabbani et al. did a cross-sectional study to evaluate the diagnostic accuracy of FAST in detecting renal injuries compared to CT. The findings indicate that FAST has a reasonable sensitivity of 85% for detecting renal injuries. This suggests that FAST is effective in identifying a significant majority of cases where renal injuries are present, making it a useful tool in trauma settings. The overall diagnostic accuracy of FAST was reported to be 83.61%. This level of accuracy indicates that FAST can be a valuable initial assessment tool in trauma patients, particularly for detecting free fluid associated with renal injuries. The study concludes that FAST can be a beneficial first-line imaging modality in trauma cases, especially in emergency settings where rapid assessment is crucial [22].

For anesthesiologists, the principles and views used in the FAST exam can be similarly applied in the postoperative period for patients who experience decompensation after abdominal surgeries. Whether in the operating room, recovery unit, or intensive care unit, elements of the FAST exam can assist providers in quickly determining if a patient's worsening hemodynamic instability is due to intra-abdominal hemorrhage or ruptured viscera. Additionally, ultrasound can swiftly identify potentially life-threatening intra-abdominal fluid and guide its drainage when necessary. To achieve sufficient tissue penetration, a low-frequency probe such as the phased array or curvilinear probe is utilized [25].

Gastric POCUS plays a crucial role in anesthesiology by assessing gastric contents, essential for reducing the risk of pulmonary aspiration during general anesthesia. Studies have shown that gastric POCUS can help identify high-risk situations for aspiration, guide anesthetic management, and optimize patient safety by determining gastric emptying and volume [26,27]. This technique is particularly valuable when patients' fasting status is uncertain or delayed gastric emptying is suspected. Additionally, gastric POCUS is reliable and accurate in various clinical settings, including pediatric anesthesia, where it can assist in confirming nasogastric tube placement and diagnosing conditions like hypertrophic pyloric stenosis. Integrating gastric POCUS into anesthesiology practice through proper training and equipment can enhance patient care and outcomes [28].

Maseri et al. did a cross-sectional survey to gather data on POCUS's clinical recognition and use among anesthesiologists in Belgium. Only 20.8% of the surveyed anesthesiologists identified the risk of a full stomach based on the patient's medical history. This indicates a significant gap in recognizing high-risk situations for gastric aspiration among practitioners. A mere 13.08% of respondents reported having received training in gastric POCUS. This highlights a critical need for enhanced educational programs to improve the knowledge and skills of anesthesiologists in this area. The survey revealed that 80.17% of participants had access to adequate ultrasound equipment. This availability is crucial for effectively implementing gastric POCUS in clinical settings. The findings underscore the importance of appropriate training and equipment to ensure gastric POCUS's safe and effective use in anesthesia practice. The study calls for additional efforts to enhance training and encourage the integration of gastric POCUS into daily clinical routines [26].

Disadvantages

POCUS in anesthesiology, while highly beneficial, does come with certain disadvantages. Challenges in low-resource settings include constrained healthcare budgets, initial equipment costs, and maintenance barriers [29]. Additionally, the vast array of applications and possibilities of POCUS can be overwhelming for novices, leading to difficulties in training and gaining proficiency. The need for ongoing quality control, standardization, and training in POCUS remains a significant hurdle, especially in ensuring minimum standards in residency programs. Despite its advantages in providing quick diagnostic data and enhancing patient outcomes, the complexity and breadth of POCUS applications require continuous education and training to maximize its potential while overcoming the associated challenges in anesthesiology [30].

Overcoming challenges

Overcoming training challenges in POCUS for anesthesiology involves standardizing training, defining credentialing/privileging protocols, and establishing billing guidelines. Anesthesiologists are integrating diagnostic POCUS into their practice to enhance patient care, necessitating a minimum level of training for competence and addressing variability in training across specialties [31]. To promote POCUS competency, anesthesiology departments should incorporate POCUS into curricula, offer training programs, and support ongoing professional development activities at a national level. The evolution of POCUS technology, its portability, and the potential for automated image analysis through artificial intelligence present opportunities to streamline training and enhance diagnostic accuracy in anesthesiology practice. By overcoming challenges through standardized training, credentialing, and ongoing education, anesthesiologists can successfully incorporate POCUS into routine practice, improving patient outcomes and diagnostic capabilities at the point of care [32]. Table 1 includes a summary of a few studies included in the study.

**Table 1:** A summary of few articles included in the study POCUS: point-of-care ultrasound; FOCUS: focused cardiac ultrasound; FAST: focused assessment with sonography for trauma; CT: computed tomography

Study	Study type	Study description	Conclusion
Hurtado et al. [8]	Literature review	The chapter synthesizes existing knowledge about POCUS in the context of regional anesthesia and airway management	Conclusions drawn from the paper emphasize the significance of POCUS in improving the safety and efficacy of regional anesthesia and airway management, advocating for individualized, less-invasive techniques while highlighting the necessity of proper training and anatomical knowledge
Arumugam et al. [9]	Literature review	The study involves an analysis of specific applications of POCUS, a discussion of its benefits and limitations, an emphasis on skill development, and a call for increased adoption among anesthesiologists. These methods collectively aim to enhance the understanding and application of POCUS in regional anesthesia and perioperative care	POCUS in regional anesthesia, highlighting its benefits in patient care, the necessity for skill development, and the potential for improved surgical efficiency. The authors encourage anesthesiologists to adopt these techniques to enhance their practice and patient outcomes
Wu and Li [10]	Observational study	The study highlights the transition from traditional methods like fiberoptic bronchoscopy to more innovative approaches like POCUS for the effective placement of bronchial blockers in children, aiming to enhance safety and efficiency in anesthetic airway management during thoracic surgeries	The study concludes that POCUS is a valuable tool for the placement of bronchial blockers in children, offering significant advantages over traditional methods and supporting its potential for broader clinical application in surgical settings
Pallesen et al. [14]	Randomized controlled trial	The study utilized a randomized controlled trial design, focused on high-risk surgical patients, implemented preoperative cardiac ultrasound as an intervention, and measured significant postoperative outcomes to evaluate the effectiveness of the intervention. This structured approach aims to provide robust evidence regarding the benefits of preoperative cardiac assessment in improving surgical outcomes	The study focused on preoperative-focused cardiac ultrasound, which may play a crucial role in improving outcomes for high-risk surgical patients by enabling tailored anesthesia and reducing the incidence of adverse postoperative events. The study highlights the need for further research to validate these findings and integrate FOCUS into standard preoperative assessments
Sheth et al. [17]	Review article	The study collected clinical and demographic information from the database. This included details such as age, gender, and the American Society of Anesthesiologists classification, which helped in understanding the patient population undergoing noncardiac surgery. The researchers identified the specific indications for performing FOCUS in patients	The paper underscores the importance of FOCUS as a transformative tool in perioperative medicine, enhancing diagnostic capabilities and management strategies for patients undergoing noncardiac surgery
Basumatary et al. [19]	Observational study	Seventy participants were included in the study. The study employed a prospective, observational design to investigate the incidence of postoperative pulmonary congestion diagnosed by lung ultrasound in patients undergoing surgeries under general anesthesia	Conclusions underscore the importance of monitoring fluid administration and surgical duration to improve patient outcomes in the postoperative setting
Rabbani et al. [22]	Cross-sectional study	The study included 139 patients, and both FAST and CT scans were performed on them. FAST is a rapid ultrasound examination used in trauma settings to detect free fluid, whereas CT is considered the gold standard for diagnosing renal injuries	The study highlights the potential of FAST as an effective initial diagnostic tool for renal injuries in trauma patients while also recognizing its limitations that warrant further investigation through more definitive imaging methods
Maseri et al. [26]	Cross-sectional survey	Methods used in this study were designed to effectively assess the knowledge, recognition, and utilization of gastric POCUS among anesthesiologists, highlighting areas for improvement in training and practice	The survey highlights critical areas for improvement in the recognition and utilization of gastric POCUS among anesthesiologists in Belgium, emphasizing the need for better training, education, and integration into clinical practice

Discussion

The incorporation of POCUS in anesthesiology has led to significant advancements in clinical practice, with various studies highlighting its effectiveness across multiple domains, including airway management, cardiac assessment, LUS, and abdominopelvic evaluations. While the benefits are clear, comparing different studies reveals both the potential and the challenges associated with the widespread use of POCUS. In airway management, studies by Hurtado et al. and Arumugam et al. emphasize the value of POCUS in enhancing the precision and safety of regional anesthesia techniques. Hurtado et al. underscore the importance of real-time visualization in improving needle placement accuracy during airway blocks, which directly translates to better patient outcomes. Similarly, Arumugam et al. highlight POCUS's broader implications in perioperative care, particularly its role in reducing surgery delays and improving operational efficiency. However, these studies also highlight the need for rigorous training to ensure that anesthesiologists can fully leverage POCUS's capabilities, as the technique's complexity may pose a barrier to novices [8,9].

In the realm of cardiac assessment, the studies by Pallesen et al. and Sheth et al. illustrate the profound impact of FOCUS on patient management, particularly in high-risk surgical patients. Pallesen et al. demonstrate that preoperative FOCUS can identify previously undetected cardiac issues, allowing for tailored anesthesia plans to improve outcomes. Sheth et al. further reinforce this by showing that FOCUS leads to management changes in a significant proportion of cases, enhancing intraoperative and postoperative decision-making. These findings collectively highlight the transformative potential of FOCUS in anesthesiology. Yet, they also underscore the necessity for proper training and the challenges of obtaining high-quality cardiac images in certain patient populations [14,17].

LUS, as examined in studies by Basumatary et al. and others, has proven to be a valuable tool for assessing pulmonary conditions, particularly in the perioperative setting. Basumatary et al. emphasize the role of LUS in monitoring intraoperative fluid management, identifying early signs of pulmonary congestion, and guiding postoperative care. The ability to detect specific lung pathologies through ultrasound artifacts further supports LUS's utility in critical care and emergency medicine. However, relying on artifact recognition rather than direct visualization presents a diagnostic challenge that requires a deep understanding of ultrasound physics and pathology, again highlighting the importance of specialized training [19].

The use of POCUS in abdominopelvic evaluations, particularly through the FAST, has become integral to trauma management, as noted in studies by Rabbani et al. and others. FAST's ability to rapidly detect intra-abdominal injuries and guide immediate interventions has been well-documented, making it a cornerstone in trauma care. The application of FAST principles in the perioperative setting, as discussed in various studies, further extends its utility to anesthesiology, where it can help manage patients with postoperative complications. However, the variability in diagnostic accuracy, as noted in the literature, underscores the need for careful interpretation of ultrasound findings and the limitations of FAST in certain clinical scenarios [22].

## Conclusions

The integration of POCUS into anesthesiology has marked a significant shift in the way anesthesiologists approach patient care. POCUS has proven a versatile and powerful tool, from enhancing the precision of airway management and guiding cardiac assessments to improving lung evaluations and abdominopelvic trauma detection. The studies reviewed demonstrate that POCUS augments traditional techniques and provides real-time, noninvasive insights that can lead to more informed and timely clinical decisions. However, the effectiveness of POCUS is closely tied to the practitioner's expertise. The benefits observed in various studies underscore the importance of comprehensive training and continuous education to ensure that anesthesiologists can fully leverage the technology's potential. Moreover, the challenges associated with image acquisition and interpretation, particularly in complex clinical scenarios, highlight the need for ongoing research and the development of standardized protocols. While POCUS has already profoundly impacted anesthesiology, its future success will depend on addressing the current training, standardization, and quality control challenges. By continuing to refine these aspects, POCUS can be further integrated into anesthetic practice, ultimately leading to better patient outcomes and more efficient perioperative care.
